# A Systematic Review of Survival Rates of Osseointegrated Implants in Fully and Partially Edentulous Patients Following Immediate Loading

**DOI:** 10.3390/jcm8122142

**Published:** 2019-12-04

**Authors:** Massimo Del Fabbro, Tiziano Testori, Vladan Kekovic, Funda Goker, Margherita Tumedei, Hom-Lay Wang

**Affiliations:** 1 Department of Biomedical, Surgical and Dental Sciences, University of Milan, 20122 Milan, Italy; funda.goker@unimi.it; 2 IRCCS Orthopedic Institute Galeazzi, Dental Clinic, 20161 Milan, Italy; info@tiziano-testori.it; 3 Department of Periodontics and Oral Medicine, The University of Michigan, School of Dentistry, Ann Arbor, MI 48109, USA; homlay@umich.edu; 4 University of Niš, 18000 Niš, Serbia; vladan.k@hotmail.com; 5 University“Gabriele D’Annunzio” of Chieti-Pescara, 66100 Chieti, Italy; margherita.tumedei@unich.it

**Keywords:** immediate loading, immediate restoration, dental implants, fully edentulous patients, partially edentulous patients, systematic review

## Abstract

Background: The aim of this systematic review was to evaluate the survival rates of immediately loaded implants after at least five years. Besides implant failure, the amount of marginal bone loss around implants and the complication type were assessed. Methods: The electronic search was undertaken on Medline, Scopus, and Cochrane Central Register of Controlled Trials using key terms such as: “immediate loading”, “immediate function”, “immediate restoration”, “immediate temporization”, “dental implants”, “fully edentulous patients”, “partially edentulous patients”. The search terms were combined using the Boolean operators AND, OR. The last electronic search was performed on 15 February 2018. Two authors independently screened the studies, extracted the data, and assessed the risk-of bias. The main outcomes recorded for each study were: implant and prosthesis success and survival, marginal bone level change, incidence and type of complications. Kaplan–Meier analysis was used to estimate cumulative survival rates. Results: Thirty-four prospective studies with at least five-year follow-up, published between 2007 and 2017 were included. A total of 5349 immediately loaded implants in 1738 patients were analyzed. The mean follow-up was 72.4 months (median 60 months, 95% confidence intervals (CI): 64.53, 80.25 months, range 60 to 147 months). The mean weighted implant survival was 97.4% (median 98.15%, 95% CI: 96.29%, 98.54%, range 83.80% to 100%). Cumulative survival rate of implants placed in the mandible was significantly higher than for the maxilla (*p* < 0.01). No significant difference in failure rate was found among the types of prosthesis employed (*p* = 0.27). The mean peri-implant bone level change at the end of the follow-up in each study ranged from 0.3 to 1.7 mm. Conclusion: Immediate loading of implants appears to have long-term predictability and success rate under well-defined circumstances.

## 1. Introduction

The original Brånemark protocol for placing dental implants required a two-stage surgery with a submerged healing period of at least three months in the mandible and six months in the maxilla, allowing the implant to osseointegrate without exposure to external trauma [1]. Under defined circumstances, early and immediate loading protocols have been recognized to be viable alternatives to the conventional one- or two-stage delayed loading approaches [2,3,4]. It was in 1977 that the timing of loading was implicated as a critical parameter governing osseointegration for the first time, by Brånemark et al. [5]. In 1977 Sarmiento et al. also provided convincing experimental evidence that early weight-bearing can accelerate the process of fracture healing [6], findings which hint at the role of the immediately loaded implants’ integration. This was in accordance with results of an earlier study by Hulbert et al. in 1974, which compared bone ingrowth in implants inserted in a weight-bearing femur and in a load-free amputated femur [7]. According to the results, bone in-growth occurred better on the weight-bearing side. The difference was mainly attributed to the presence of stress exerted during healing [7]. Rubin and McLeod also reported that brief exposure to mechanical strains might enhance the biological fixation of implants [8]. Consequently, the desire for fewer surgical interventions and shorter implant treatment times have led to the development of revised placement and loading protocols [9,10]. Among the most innovative procedures introduced, immediate loading stands out for its by now routine clinical applications [11,12].

Two types of immediate loading have been described in the literature. One is the immediate functional loading (IFL), or immediate occlusal loading, which refers to the use of a temporary or definitive prosthesis seated the same day as the surgery in occlusal contact with the opposing arch [13]. An alternative approach consists in modifying the immediate temporary restoration to avoid occlusal contacts in centric and lateral excursions, in order to reduce the early risks of mechanical overload caused by functional or parafunctional forces, the immediate nonfunctional loading (INFL), or immediate nonocclusal loading. Thus, the modified restoration would still be involved in the masticatory process, but the mechanical loading stress is reduced [10,14]. Parameters such as flap or flapless surgery are also considered, as flapless surgery plays an important role in avoiding additional bone resorption from the bony surface related to the elevation of the mucoperiosteal flap [15]. The success of an implant is evaluated by taking into account numerous factors, such as maintenance of function, stability, lack of signs and symptoms, absence of peri-implant radiotransparency, limited loss of marginal bone, and health of peri-implant soft tissues.

The purpose of the present systematic review is to investigate the prognosis of immediately loaded implants, through assessment of implant and prosthesis survival and success rates after at least five years of functional loading. Furthermore, the amount of marginal bone loss around implants, the type and incidence of complications, and the occurrence of implant failures were assessed to determine if immediate loading of implants can be the treatment of choice under well-defined circumstances.

## 2. Materials and Methods

The present review was undertaken by following the Preferred Reporting Items for Systematic Reviews and Meta-Analyses (PRISMA) guidelines [16].

### 2.1. Statement of Question (PICOS)

A PICO question was devised to identify the objectives and the inclusion criteria where, P is, population or patient, I, intervention, C, comparisons, O, outcomes, and S stands for study design. When the aim of a systematic review is to determine the efficacy of an intervention, only randomized controlled trials must be searched. The present review aimed at evaluating the prognosis of immediate loading, independent of comparison with conventional delayed loading, which has a well-established prognosis. In other words, it was not mandatory to select only comparative clinical trials, randomized or not. The PICO question was: “In partially and fully edentulous patients, what are the implant and prosthesis survival rates, the incidence of complications and the marginal bone level changes after a minimum of five years in patients treated with immediately loaded implants, reported by prospective clinical trials?”.

### 2.2. Search Strategy

A comprehensive electronic search was undertaken on Medline, Scopus, and Cochrane Register of controlled trials, to identify prospective clinical studies reporting the main outcomes (implant/prosthesis survival/success rate, marginal bone loss, biological and mechanical complications) of immediately loaded implant-supported restorations. The search was conducted using the following search string, composed of key terms combined with the Boolean operators AND, OR: ((immediate loading OR immediate function OR immediate restoration OR immediate temporization) AND dental implants AND ((fully OR completely OR partially) AND edentulous) AND (patients OR arch OR site OR jaw OR mandible OR maxilla)). The references of the selected articles and previous reviews were also examined for identifying further eligible studies. The last electronic search was performed on 15 February 2018.

### 2.3. Inclusion Criteria

The studies to be included in this systematic review had to meet the following inclusion criteria:

- Human studies.
- Publication in English language.
- Prospective studies (randomized clinical trials (RCT), controlled clinical trials (CCT) or prospective case series (PCS)).
- Functional fixed prosthesis delivered within 72 h after postimplant placement.
- At least five years follow-up after prosthesis delivery.
- At least 10 patients treated with immediately loaded implants.
- Patients older than 18 years.
- Data regarding success and/or survival of immediately loaded implants, as well as complications, had to be reported.
- No restriction was placed regarding the publication year.
- Case reports, retrospective studies, and reviews of the literature, as well as animal and in vitro studies, were excluded.
- When papers from the same group of authors were identified, with very similar databases of patients, materials, methods, and outcomes, the authors were contacted to clarify whether the pool of patients was indeed the same. In the case of multiple publications relative to different aspects or phases of the same study, only the one reporting results with the longest follow-up was considered.

### 2.4. Selection Criteria and Data Extraction

Firstly, titles and abstracts of articles identified through electronic search were screened independently by two reviewers (F.G., V.K.) to exclude irrelevant papers and non-prospective studies, and to select articles fulfilling the inclusion criteria. When a decision could not be made based on the title and abstract, the full text was obtained and assessed, and a third reviewer was involved (MDF). For all eligible studies, the full text was obtained and analyzed in order to confirm that the studies met the inclusion criteria and determine the list of articles included in the review. For all studies excluded at this stage, reasons for exclusion were provided.

Data from included studies were independently extracted by two reviewers (F.G. and V.K.). Cases of disagreement were subject to joint evaluation by the reviewers until an agreement was reached. A third reviewer (MDF) was consulted if needed.

The following information was extracted from each included study and recorded using a specially designed data sheet: year of publication; study design; study setting (university, hospital or private); sample size calculation (yes/no); blinding of evaluators (yes/no); type of fixation (cemented or screwed); number of patients, number of implants, number of implants per patient, number of male or female patients, mean age and range, number of smokers, number of postextraction immediately loaded implants, location (implants placed in the anterior, posterior or both regions), jaw, time of loading (same day, within 48 h, within one week), type of loading (occlusal or non-occlusal), type of prosthesis (single tooth, fixed partial prosthesis or fixed full prosthesis), type of definitive prosthesis retention (screw retained or cemented), implant brand and type, surface type, torque levels, flap or flapless approach, patients demographics (age, gender, number of smokers), mean follow-up duration, number of dropouts, reason and time of failures, marginal bone level changes after one year and after five years of follow up, soft tissue changes, aesthetic evaluation, and type and number of complications.

### 2.5. Methodological Quality Assessment

The quality of the included studies was assessed by means of the Newcastle–Ottawa Scale (NOS) for nonrandomized clinical studies (case-control and cohort studies). The scale is a star system based on three domains: 1. Selection of study groups (four items, up to four stars); 2. Comparability of the groups (one item, up to two stars); 3. Ascertainment of either the exposure or outcome of interest for case-control or cohort studies, respectively (three items, up to three stars). Stars are awarded so that the highest quality studies are assigned up to nine stars.

### 2.6. Data Analysis

Data were tabulated and analyzed using the software Microsoft Excel 2016 (© 2016 Microsoft Corporation, Santa Rosa, CA, USA) and the software GraphPad Prism 5.0 (GraphPad San Diego, CA, USA). For the data synthesis, weighted mean values, median, 95% confidence intervals and ranges were used. Distribution of implant failures was assessed using a time-to-event analysis. Studies that did not provide information regarding the timing of implant loss were excluded from the analysis. Life table analysis and Kaplan–Meier analysis were used to estimate cumulative implant survival rate. The cumulative survival rates of implants in the maxilla and mandible were compared using log-rank (Mantel–Cox) test. The significance threshold was set at *p* = 0.05.

## 3. Results

**Figure 1:**
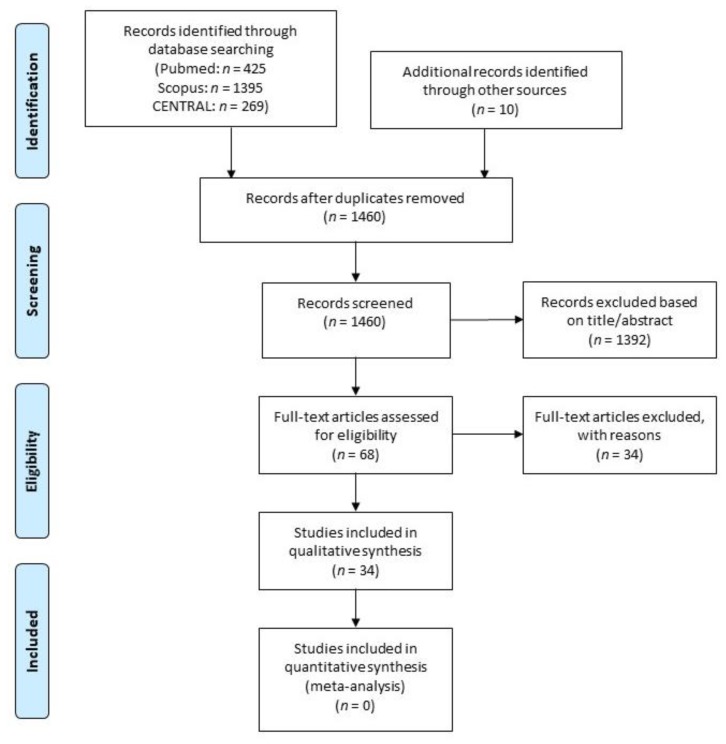
PRISMA flow chart of the study selection process.

The search results and the flow of study selection is shown in Figure 1. The included studies are reported in Table 1. A total of 34 studies presenting the results of immediately loaded rehabilitations with at least five-year follow-up were analyzed [17,18,19,20,21,22,23,24,25,26,27,28,29,30,31,32,33,34,35,36,37,38,39,40,41,42,43,44,45,46,47,48,49,50]. Complete prostheses, partial prostheses, and single crowns were considered. Both implants placed in healed sites and in fresh postextraction sites were included. Collectively, these studies, published between 2007 and 2017, reported on 5349 immediately loaded implants in 1738 patients (on average, 3.08 implants per patient). The mean follow-up was 72.4 months (median 60 months, 95% confidence intervals (CI): 64.53, 80.25 months, range 60 to 147 months).

**Figure 2:**
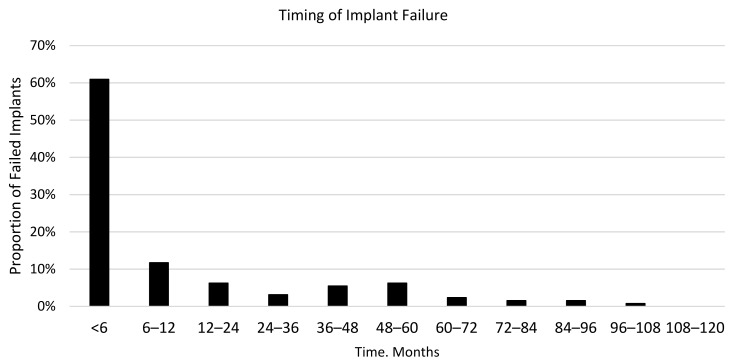
Proportion of the failed implants according to the timing of failure.

A total of 135 implant failures was reported. Most failures occurred early after loading or during the first year. In particular, 60.9% of failures occurred within the first six months and 75.0% in the first year, as seen in Figure 2.

The overall mean weighted implant survival was 97.41% (median 98.15%, 95% CI: 96.29%, 98.54%, range 83.80% to 100%). Cumulative implant survival up to five and ten years follow-up was 97.66% and 96.94%, respectively, as seen in Table 2. Only one study did not provide information regarding the timing of failure [24] and was excluded by the life table and the Kaplan–Meier analysis.

**Figure 3:**
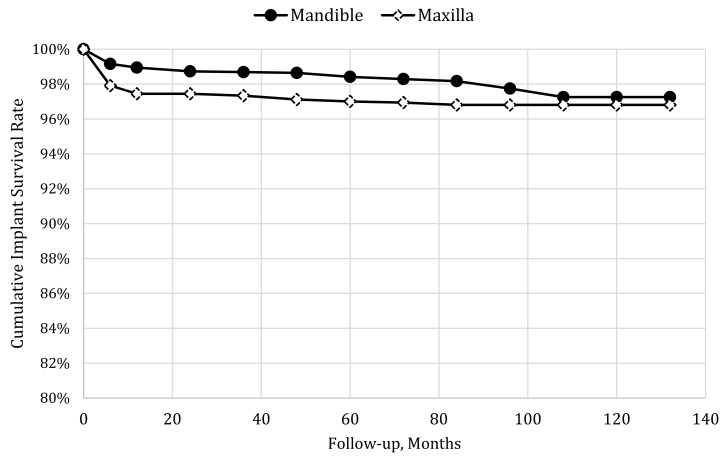
Kaplan–Meier analysis for mandible and maxilla.

In the mandible, cumulative implant survival at five and ten years follow-up was 98.42% and 97.26%, respectively, while in the maxilla, it was 97.01% and 96.81%, respectively, as seen in Figure 3. The difference in cumulative implant survival rate between maxilla and mandible up to five and ten years follow-up, estimated by log-rank test, was significant (*p* = 0.0008 and *p* = 0.0027, respectively), i.e., immediately loaded implants placed in the maxilla tended to fail more than those placed in the mandible.

Mean weighted implant survival for single tooth, partial fixed prosthesis and full-arch fixed prosthesis was, respectively, 96.19% (median 98.55%, 95% CI: 92.70%, 99.69%, range 80% to 100%), 98.51% (median 98.60%, 95% CI: 97.39%, 99.63%, range 95.50% to 100%) and 96.71% (median 99.05%, 95% CI: 92.71%, 100.7%, range 65.70% to 100%). No significant difference in failure rate was found regarding prosthesis type (*p* = 0.27). For three studies in which multiple prosthesis types were used, it was not possible to split implant survival data based on prosthesis type [20,23,30].

The mean peri-implant bone level change at the end of the follow-up in each study ranged from 0.3 to 1.7 mm, with no significant difference between the two jaws.

The majority of studies reported only “minor” or “nonserious” complications, meaning technical and prosthetic issues (e.g., screw loosening, crown decementation, porcelain fracture) that were easily resolved chairside. Biological complications, consisting of peri-implantitis and peri-implant mucositis, were less frequently reported, but were often associated with late implant loss.

The results of the methodological quality assessment are reported in Table 3. Most studies (24 out of 34) were scored 4 to 5, only one study was scored 7 and one 8, indicating a general poor to medium methodological quality of the included studies, very few studies were judged to be of good to excellent quality.

## 4. Discussion

Among the innovative procedures marking significant steps forward in implant dentistry, immediate loading stands out for its importance in routine clinical practice [51]. Under defined circumstances, early and immediate loading protocols are now deemed viable alternatives to the conventional one- or two-stage delayed loading approaches [2,3,52]. Indications for immediate loading, well-documented over the years, range from implant placement in the fully edentulous mandible and maxilla to single tooth applications in extraction sockets [53]. It is assumed that immediate loading of implants may have a positive influence on implant therapy outcomes as there is proof that presence of functional biomechanical stimuli exerted during healing enhances the biological fixation of implants [8]. In immediate loading, two modalities are utilized in the temporization phase: functional loading, which stands for implant prosthesis being seated at the time of implant placement and immediately subjected to functional occlusal loading, and nonfunctional loading, in which implants are immediately loaded but prosthesis is kept out of direct occlusal contact. In the latter, a certain amount of loading occurs from lip and tongue pressure and contact with food, but not from contact with the opposing teeth.

According to the recommendations of ITI Consensus Statement in 2014 [54], the definition and classification of immediate loading was settled as follows; (i) Conventional loading of dental implants is defined as being greater than two months subsequent to implant placement. (ii) Early loading of dental implants is defined as being between one week and two months subsequent to implant placement. (iii) Immediate loading of dental implants is defined as being earlier than one week following implant placement [54,55].

Achievement of adequate stability depends on controlling micromovements in the interface between the implant and bone [12], as each loading regimen induces a different mechanical environment that is, depending upon implant design, converted into a distinct magnitude of motion at the implant–bone interface [52].

A trending question in the field of implantology regards marginal bone loss that occurs around implants. Recent reviews and consensus papers state that, besides peri-implant infection, there may be various other reasons for the loss of marginal bone [9]. These include physiological remodeling after placement, occlusal overload, quality of surgical and prosthetic treatment, quality of oral hygiene, and systemic disease [33]. Furthermore, the peri-implant mucosa needs to be supported by an adequate three-dimensional (3D) osseous volume of the alveolar ridge [56]. Especially, when replacing teeth in the anterior zone, particular attention should be paid to the aesthetic outcome [57].

The ultimate goal of immediate loading protocol is to reduce the number of surgical interventions and to shorten the time frame between surgery and prosthesis delivery, all without compromising the success rate of the procedure. Immediate temporization of implants has been introduced to meet many needs, including the high survival rates of implants and prosthetic restorations, the preservation of marginal bone levels, and the satisfaction of patients. This leads to the primary objectives of this paper: (a) assessing the survival rates of immediately loaded implants after a minimum of five years of function, (b) assessing the survival rates of the prosthetic restorations, and (c) assessing the levels of marginal bone loss around these implants after one year and five years.

These protocols will ultimately lessen patients’ reservations, resulting in increased acceptance of implant therapy [58]. Various indications for immediate loading have been discussed; they range from implant placement in the fully edentulous mandible and maxilla to single tooth applications in extraction sockets [53].

Bone preservation is a key factor for aesthetic outcome [59] as supracrestal tissues closely follow the changes of the underlying bone [60]. The presence of papillae is primarily related to the bone level at the adjacent tooth. With this in mind, secondary objectives of this article are related to the soft tissue aesthetic scores in immediately loaded implants. Parameters such as flap or flapless surgery were also considered, as flapless surgery plays an important role in avoiding additional bone resorption from the bony surface caused by mucoperiosteal flap elevation [15,59].

Functional, biological, and aesthetic considerations need to be made for achieving predictable long-term tissue stability [61]. Peri-implant soft tissue preservation is related to many clinical parameters [62]. Recent reviews and consensus papers state that, besides peri-implant infection, there may be various other reasons for the marginal bone loss [9]. These include physiological remodeling after placement, occlusal overload, quality of surgical and prosthetic treatment, quality of oral hygiene, and systemic disease [33].

Evidence-based medicine aims to provide patients with the best possible treatment by integrating the clinician’s skill with the best available scientific evidence from the literature, and by taking patients’ preferences and needs into consideration. Today, patients are no longer considered inert subjects who passively undergo the doctor’s decisions. Rather, they actively and knowledgeably participate in the decision-making process regarding their treatment.

As a result of this shift in patient–clinician relations, it has become evident that treatment outcomes need to be assessed through patient-based parameters, with the patient becoming central in the overall analysis. Involvement of the patient in treatment outcome assessment is becoming more and more common. It is no longer sufficient to claim the treatment a success merely based on clinical and technical aspects. Conversely, it is necessary that the patient is satisfied with as many aspects as possible, which provides not only complete restoration of function and aesthetics, but also psychological well-being. Immediate loading not only demonstrates a high long-term predictability, but appears to be able to meet all of the above aspects addressing the patient’s needs. In modern implant dentistry, immediate loading should become routine treatment. Nevertheless, it is just as important not to forget that such a treatment can bring to excellent levels of satisfaction, but must only be applied when the fundamental clinical requirements are satisfied. In fact, any abuse or misuse of immediate loading might increase the risk of failure, with biological and psychological consequences for the patients. The distribution of the included articles over the years shows that there is a growing interest in immediate loading, allowing more insight into the most adequate clinical protocols, favoring the integration of immediate implant loading into everyday practice.

## 5. Conclusions

In conclusion, the results of this review confirm that immediate implant loading is a predictable protocol that can be the therapy of choice under appropriate circumstances, leading to excellent, long-lasting favorable outcomes and high patient satisfaction.

## Figures and Tables

**Table 1:** Main features of the included studies.

Reference, Year	Study Design	Prosthesis Type	No. Total IL Patients	Complications/Time	No. IL Implants MAND/MAX	Follow-Up, Mean (Range)	Implant Loss and Time	Implant Survival Rate %	Bone Loss, mm (Mean ± SD)
Jaffin et al. 2007 [17]	PCS	Fixed Full	17	None reported	139 (54 mand/85 max)	60 months	No failures	100.0%	NR
Calandriello et al. 2009 [18]	PCS	Single tooth	33	None reported	40 mand	60 months	Two failures in mand; three and ten months	95.0%	1.17 ± 0.90
Degidi et al. 2009 [19]	RCT	Fixed Full/Single tooth	82	None reported	262 (167 mand/73 max/22 single) + 286 control	60 months	Three failures in mand; five years	98.86%	0.9
Payer et al. 2010 [20]	PCS	Fixed partial/Single tooth	24	1 comp/8 week	40 mand	60 months	Three failures in mand; 12 months	92.50%	1.21 ± 1.12
Capelli et al. 2010 [21]	RCT	Fixed partial/Single tooth	25	1 comp/33 month	52 (38 mand/14 max)	60 months	One failure; two months	98.08%	1.18 ± 0.56
Prosper et al. 2010 [22]	PCS	Single tooth	35	None reported	60 mand	60 months	Two failures; 60 months	96.67%	1.31 ± 0.44
Malchiodi et al. 2010 [23]	PCS	58 Fixed Full/70 Fixed partial/30 Single tooth	70	Not Reported	158 max	60 months	Two failures max; 23–26 days	98.73%	NR
Balshi et al. 2011 [24]	PCS	Single tooth	140	Not Reported	164 (28 mand/136 max)	66 months	Three mand/four max failures (time NR)	95.73%	NR
Malo et al. 2011 [25]	PCS	Fixed Full	245	1 biol. comp/4 mo 12 mech. comp	980 mand	10 years	Twenty-one failures (different times)	97.86%	NR
Özkan et al. 2011 [26]	PCS	Twelve single tooth/36 Fixed partial	28	Four porcelain fractures	84 max	60 months	No failures	100%	0.34
Mertens et al. 2011 [27]	PCS	Four fixed full/14 fixed partial/31 single tooth	17	None reported	14 (5 mand/9 max)	60 months	No failures	100%	0.1 ± 0.4
Horwitz et al. 2012 [28]	PCS	Fixed full mouth/fixed partial	19		74 (28 mand/46 max)	60 months	Twelve failures before six months	71.43%	1.41 ± 0.67
Levine et al. 2012 [29]	PCS	Single tooth	20	One crown decementation	21 mand	60 months	No failures	100%	0.58
Degidi et al. 2013 [31]	PCS	Fixed full	52	One peri-implantitis/ 25 mucositis	256 (144 max/112 mand)	72 months	Two max (<6, 60–72 months), one mand (<6 months)	98.8%	1.39 (Max) 1.29 (Mand)
Romanos et al. 2013 [32]	PCS	Fixed full	20		163	80.3 months	Three failures; four months (max nonsmoker), eight months (mand. smoker), 78 months (max smoker)	98%	0.46 ± 0.98(Sm) 0.43 ± 1.35(NSm)
Davó et al. 2013 [30]	PCS	Fixed full/fixed partial	42	Swelling, pain	221 max (2 zy)	60 months	Nine failures (eight <6 months, one at 36–48 months)	95.93%	NR
Glauser et al. 2013 [33]	PCS	Twenty single tooth/one fixed full/30 fixed partial	38	“Nonserious compl.”	102 (38 max/64 mand)	61.3 months	Three max failures; <3 months	97.10%	1.54
Rocci et al. 2013 [34]	RCT	Fixed partial	22	None reported	66 mand	9 years	Three failures; <7 months	95.5%	0.9
Tealdo et al. 2014 [35]	CCT	Fixed Full	34	None reported	163 max	75.2 months	Ten failures; <3 months	93.9%	1.62 ± 1.12
Crespi et al. 2014 [36]	RCT	Fixed Full	28	“Minor compl.”	272 (192 max/80 mand)	84 months	Two failures, no region; two months	99.27%	0.32 ± 0.21 (CR) 0.48 ± 0.40 (SR)
Cooper et al. 2014 [37]	PCS	Single tooth	94	Minor papilla problems	113 max	60 months	Four failures; <1 year	96%	0.43 ± 0.63 (FES) 0.38 ± 0.62 (HR)
Jokstad et al. 2014 [38]	RCT	Fixed full	16	None reported	64 mand	60 months	No failures	100%	1.3 ± 0.7
Donati et al. 2015 [39]	RCT	Single tooth	104	“Minor compl.”	111 anterior	60 months	Four failures before three months	97.10%	0.27
Shigehara et al. 2015 [40]	PCS	Fixed full	27	“Minor compl.”	189	77.9 months	No failures	100.0%	NR
Romanos et al. 2014 [41]	RCT	Fixed partial	13	None reported	78 mand	12.27 years	No failures	100%	0.70 ± 1.09 (Mes) 0.43 ± 1.02 (Dis)
Toljanic et al. 2016 [42]	PCS	Fixed full	51	“Minor compl.”	306 max	60 months	Twenty failures; two years	92%	0.44 ± 1.25
Cannizzaro et al. 2016 [43]	PCS	Fixed full	79	“Minor compl.”	158 mand	60 months	Two failures; three weeks	98.70%	0.69
Canullo et al. 2016 [44]	RCT	Single tooth	22	None reported	22 max	10 years	No failures	100%	0.49 ± 0.27
Glibert et al. 2016 [45]	PCS	Nineteen single tooth/23 fixed partial/eight fixed full	40	Not reported	112 (40 mand/72 max)	6.2 years	One failure at three months	99.10%	0.35
Tallarico et al. 2016 [46]	RCT	Fixed full	40	“Minor compl.”	200 max	60 months	Seven failures; five at <6 months, two at 24–36 months	97.50%	1.71 ± 0.42 (Ao4) 1.51 ± 36 (Ao6)
Agliardi et al. 2017 [47]	PCS	Fixed full	15	“Minor compl.”	60 max (42 zy)	79 months	No failures	100%	1.39 ± 0.10
Garlini et al. 2017 [48]	PCS	Fixed partial	94	1 suppuration	147 (41 mand/106 max)	10 years	Two failures; <1 month	98.56%	NR
Meloni et al. 2017 [49]	PCS	Fixed full	66	Minor or technical	356 (92 mand/264 max)	71.2 months	Five failures in 0–1 years, two failures in 3–5 years	98%	1.61 ± 0.41
Raes et al. 2017 [50]	PCS	Fixed partial	96	“Minor compl.”	102 (single ant max)	60 months	Two failures; 6–12 months	98%	NR

IL—immediate loading; PCS—prospective clinical study; CCT—controlled clinical trial; RCT—randomized clinical trial; zy—zygomatic implants; CR—cement-retained; SR—screw-retained; NR—not reported; FES—fresh extraction socket; HR—healed ridge; Mes—mesial; Dis—distal. Sm—smokers; NSm—non-smokers; Ao4—all-on-four; Ao6—all-on-six.

**Table 2:** Life table analysis of the overall data.

Interval, Months	Implants at Risk	Failed Implants	Dropouts/Lost to Follow-Up	Implant Survival Rate	Cumulative Survival Rate
0–6	5163	78	24	98.49%	98.49%
6–12	5061	18	55	99.64%	98.14%
12–24	4991	5	66	99.90%	98.04%
24–36	4917	4	62	99.92%	97.96%
36–48	4851	7	61	99.86%	97.82%
48–60	4783	8	1644	99.83%	97.66%
60–72	3131	3	1598	99.90%	97.56%
72–84	1530	2	491	99.87%	97.43%
84–96	1037	2	715	99.81%	97.25%
96–108	320	1	76	99.69%	96.94%
108–120	243	0	67	100.0%	96.94%
>120	176	0		100.0%	96.94%

**Table 3:** Scores of the Newcastle–Ottawa scale for assessing the quality of the included studies.

Reference, Year	Selection 1	Selection 2	Selection 3	Selection 4	Comparability	Outcome 1	Outcome 2	Outcome 3	Total
Jaffin et al. 2007 [17]							*	*	2 *
Calandriello et al. 2009 [18]							*	*	2 *
Degidi et al. 2009 [19]				*		*	*	*	4 *
Payer et al. 2010 [20]	*		*			*	*		4 *
Capelli et al. 2010 [21]	*	*		*	*		*	*	6 *
Prosper et al. 2010 [22]			*			*	*	*	4 *
Malchiodi et al. 2010 [23]			*			*	*	*	4 *
Balshi et al. 2011 [24]		*	*				*		3 *
Malo et al. 2011 [25]			*			*	*		3 *
Özkan et al. 2011 [26]			*	*		*	*	*	5 *
Mertens et al. 2011 [27]			*	*		*	*	*	5 *
Horwitz et al. 2012 [28]			*			*	*	*	4 *
Levine et al. 2012 [29]			*			*	*	*	4 *
Degidi et al. 2013 [31]				*			*	*	3 *
Romanos et al. 2013 [32]			*			*	*	*	4 *
Davó et al. 2013 [30]			*			*	*		3 *
Glauser et al. 2013 [33]			*			*	*	*	4 *
Rocci et al. 2013 [34]	*	*			*	*	*	*	6 *
Tealdo et al. 2014 [35]			*	*		*	*	*	5 *
Crespi et al. 2014 [36]			*		**	*	*	*	6 *
Cooper et al. 2014 [37]			*	*			*	*	4 *
Jokstad et al. 2014 [38]	*	*	*		**	*	*	*	8 *
Donati et al. 2015 [39]			*		*	*	*		4 *
Shigehara et al. 2015 [40]			*			*	*	*	4 *
Romanos et al. 2014 [41]			*		*	*	*	*	5 *
Toljanic et al. 2016 [42]			*	*		*	*	*	5 *
Cannizzaro et al. 2016 [43]			*		*	*	*	*	5 *
Canullo et al. 2016 [44]	*	*	*		*	*	*	*	7 *
Glibert et al. 2016 [45]				*		*	*	*	4 *
Tallarico et al. 2016 [46]	*		*			*	*	*	5 *
Agliardi et al. 2017 [47]			*			*	*	*	4 *
Garlini et al. 2017 [48]			*	*			*	*	4 *
Meloni et al. 2017 [49]			*			*	*	*	4 *
Raes et al. 2017 [50]			*			*	*	*	4 *

* High quality scores are identified with a star. The maximum score is nine stars. Explanation of codes: Selection: (1) representativeness of the exposed cohort (one star); (2) selection of the non-exposed cohort (one star); (3) ascertainment of exposure (one star); (4) demonstration that outcome of interest was not present at start of study (one star); Comparability: comparability of cohorts on the basis of the design or analysis (up to two stars); Outcome: (1) assessment of outcome (one star); (2) was follow-up long enough for outcomes to occur (one star); (3) adequacy of follow up of cohorts (one star).
